# Supplementary figures and images for: Selective Encapsulation of the Polyphenols on Silk Fibroin Nanoparticles: Optimization Approaches
Source: Int J Mol Sci. 2023 May 26;24(11):9327. doi: 10.3390/ijms24119327 (PMC10252937; doi:10.3390/ijms24119327)

## Supplementary data

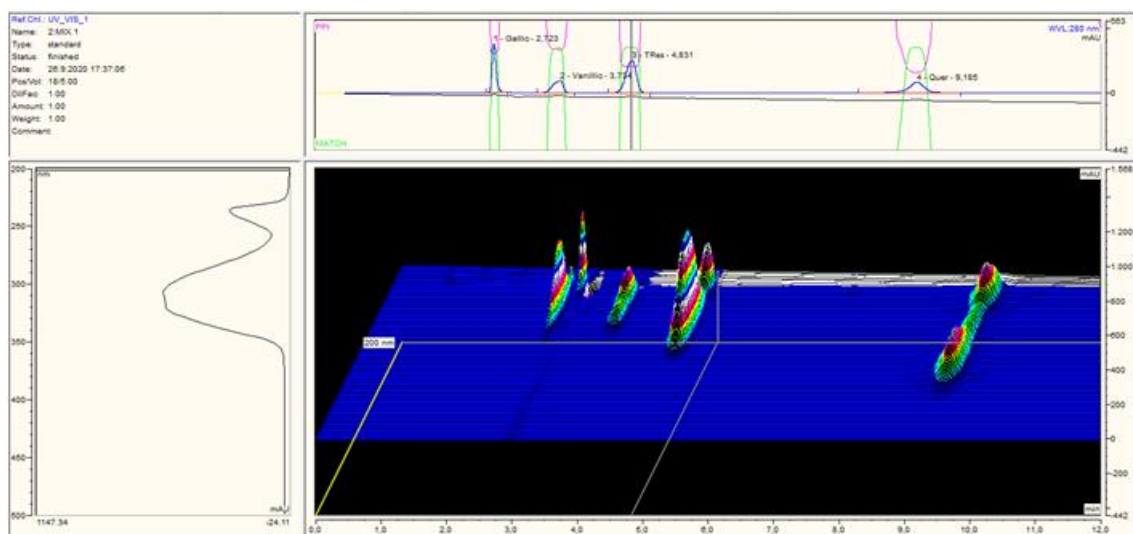

Figure S1. 3D chromatogram of the phenolic compounds of PFmix.

Supplement: Supplementary file 1 [file ijms-24-09327-s001.zip › ijms-2371354-supplementary.pdf]
